# Retraction Note: SIRT6 drives epithelial-to-mesenchymal transition and metastasis in non-small cell lung cancer via snail-dependent transrepression of KLF4

**DOI:** 10.1186/s13046-023-02759-1

**Published:** 2023-07-15

**Authors:** Ziming Li, Jia Huang, Shengping Shen, Zhenping Ding, Qingquan Luo, Zhiwei Chen, Shun Lu

**Affiliations:** Shanghai Lung Cancer Center, Shanghai Chest Hospital, Shanghai Jiao Tong University, Shanghai, China

**Retraction Note:** ***J Exp Clin Cancer Res*** **37, 323 (2018)**

10.1186/s13046-018-0984-z

The Editor-in-Chief has retracted this article on the corresponding author’s request after becoming aware of inconsistencies in the data set. Specifically, data presented in Figs. 2c and 3f appear to derive from data in [1] by different authors. Further checks by the publisher identified an additional concern about the similarity in the GAPDH controls in Fig. 4d. The authors stated that they no longer have access to the raw data. Therefore, the Editor has lost confidence in the data presented in the article. Zhiwei Chen, Shun Lu, Qingquan Luo, Jia Huang and Ziming Li have agreed to this retraction but not to the wording of this retraction notice. Zhenping Ding and Shengping Shen have not responded to any correspondence from the editor/publisher about this retraction.
